# ‘Building our own house’ as an insider-only Community-Partnered Participatory Research Council: Co-creating a safe space for Autistic knowledge production

**DOI:** 10.1177/13623613241253014

**Published:** 2024-05-17

**Authors:** Gemma L Williams, Rebecca Ellis, Willow Holloway, Selena Caemawr, Monique Craine, Kathryn Williams, Aimee Grant

**Affiliations:** 1 Swansea University, UK; 2 Autistic UK, UK; 3 Fair Treatment for the Women of Wales, UK; 4 Aubergine Café, UK; 5 NeuroDivergent Matters, UK; 6 Cardiff University, UK

**Keywords:** action research, autism, community-based participatory research, participatory action research, participatory research, qualitative research

## Abstract

This article describes and reflects on the first six meetings of a newly established Autistic community council, founded to steer a participatory, longitudinal qualitative study investigating the reproductive health of Autistic people with wombs. The two key aims of these initial six meetings – involving four Autistic community leader members and three Autistic researchers – was to (1) guide the start of the project and the development of accessible recruitment materials for the future eight lay members and (2) establish a safe space and a model of accessible communication. We draw on extracts from transcripts made during meetings, member ‘field’ notes and a reflexive co-writing practice to identify the ways in which a sense of safety was built during these first community council meetings. We explore what it means to be an *insider-*only Community Partnered Participatory Research Council and reflect on the praxis we developed to facilitate Autistic knowledge production.

**Lay Abstract:**

In recent years, there has been a growing call for *participatory Autism research* (i.e. research that meaningfully involves Autistic people in its design and delivery). *Community Partnered Participatory Research* is a research methodology that aims to share power between researchers and members of the researched community. There is some precedent for Community Partnered Participatory Research in Autism research, but it is still quite uncommon. At the start of our new research study (called Autism: From Menstruation to Menopause), we created a community council. For the first six meetings, our council was made up of four Autistic community members who were experienced in Autism advocacy and activism and three Autistic researchers. We seven are the authors of this article. In these first six meetings, we made plans for recruiting a larger number of lay community members who would join us later for the rest of the project (8 years in total). In this article, we describe and reflect what it felt like during these first six meetings to be part of a community research council where everybody is Autistic. We discuss how we co-created a safe space, how we helped each other feel valued and how we worked together to support each other’s sometimes-differing access needs so that everyone could fully participate. We provide recommendations for how to support Autistic people to lead research on their own terms with their unique insights.

## Introduction: Autistic authority and autonomy in research

Over recent years, there has been an increasing call (albeit from a minority) for participatory autism research (see, e.g., Fletcher-Watson et al., 2019; Nicolaidis et al., 2019). Participatory methodologies aim to democratise research practices and redistribute power from the researcher to the researched community (Kara, 2017; Nicolaidis et al., 2019). Participatory methodologies are not only ethical but also an epistemological matter (i.e. they can shape how we conceptualise ideas), as Pellicano (2020, pp. 233–234) explains:

Involving people who draw on their own lived experience to help us think outside the ‘normative’ box could also have far-reaching and disruptive effects on basic autism science.

Walker (2019, p. 44) takes this further:

. . . the bulk of the misguided theories and harmful practices around autism that have been generated within the pathology paradigm seem to originate in misinterpretations of the surface behaviours of autistics, based in a lack of awareness of these factors and lack of understanding of subjective autistic experience.

It is arguable that the *double empathy problem* (see the work of Milton et al., 2018) – that is, difficulties experienced by both Autistic and non-Autistic people in understanding the behaviours and communication of those from another neurotype – has a role to play in how Autistic ways of being have often been (sometimes gravely) misinterpreted by non-Autistic researchers. *Autistic expertise* (Milton, 2014) grounded in lived experience and implicit, ‘insider’ knowledge may be a remedy for this. However, even when Autistic involvement *is* sought in research, it can often feel tokenistic:

Were we there because they valued our input, or were we tokens, useful for ‘authenticity’ and snippety quotes, to validate the researcher’s inclusive credentials?

. . . my point of view was cherry picked for the bits that enhanced the project, omitting anything that questioned it. (Michael, 2021, pp. 118–119)

For participatory research to be meaningful, involvement must permeate the whole process: across research design, delivery, evaluation and dissemination (Fletcher-Watson et al., 2019; Pukki et al., 2022). *Community-Based Participatory Research* (CBPR)^1^ – ‘where community members and academics collaborate as equal partners to conduct research for improving health and wellbeing through action’ (Nicolaidis and Raymaker, 2015, p. 151) – sets the highest standards for community involvement, built around nine guiding principles (see Box 1).

**Box 1.** Principles.

CBPR/CPPR . . . 1. . . . acknowledges community as a unit of identity. 2. . . . builds on strengths and resources within the community. 3. . . . facilitates a collaborative, equitable partnership in all phases of the research. 4. . . . fosters co-learning and capacity building among all partners. 5. . . . integrates and achieves a balance between knowledge generation and intervention for the mutual benefit of all partners. 6. . . . focuses on the community relevance and on ecological perspectives that attend to the multiple determinants of health and wellbeing. 7. . . . involves systems development using a cyclical and iterative process. 8. . . . disseminates results to all partners and involves them in the wider dissemination of results. 9. . . . involves a long-term process and commitment to sustainability.

Source: Derived from Israel et al. (2001).

Collaboration between academics and community members integrated within research teams is essentially an unequal relationship because ‘the question of what counts as knowledge and whose knowledge counts are fundamentally crossed by questions of power and privilege’ (Rose & Kalathil, 2019, p. 2). Accordingly, in CBPR/CPPR (Community Partnered Participatory Research) projects, some form of negotiation is always required between different ways of knowing, measuring and valuing knowledge and between what might be best described as *outsider* (i.e. usually non-Autistic, academic researchers) and *insider* (Raymaker, 2017, usually non-academic, Autistic) positionalities. This distinction, of course, is not a hard-and-fast rule as the line between *outsider* and *insider positionalities* is increasingly blurred, particularly as the number of Autistic *insider researchers* grows. However, situated as it is within a neuronormative research paradigm, coproduction approaches aim to create a space in which ‘the expert knowledge of the professional and the expert experience of [community members]’ can coexist (Rose & Kalathil, 2019, p. 2). Yet even in the most cooperative CBPR/CPPR projects, the balance lies in favour of the (often majority non-Autistic) academic partners as knowledge production is framed through a neurotypical, academic lens (Botha & Cage, 2022).

## The Autism from Menstruation to Menopause Study Community Council

To address the knowledge gap in Autistic reproductive health (see, e.g., Williams et al., in press), our longitudinal, participatory, qualitative research project was developed in collaboration with the Autistic community to run from 2022 to 2030. The overall aim of this project is to understand Autistic reproductive experiences throughout the life course with the view to informing improved healthcare services and thus reducing the inequity many Autistic people with wombs face^2^ (Carneiro, 2023). An Autistic community council was established at the inception of the project, comprising four Autistic community leaders (established advocates and activists) and eight Autistic lay members, supported by four Autistic researchers. The aim of this article is to explore the ways in which our wholly Autistic team used the principles of CBPR/CPPR. This includes reflecting on how being an entirely insider team fostered community connectedness, and what this might mean for neurodivergent knowledge production. This aim was generated during an early meeting of the council, when council members noticed particularities in the ways our group interactions were unfolding (compared with our experiences in other groups of mixed neurotypes) and the emergence of a distinct, collective, communicative praxis:

S.C.: It would be really interesting if somebody could literally just go through all of these meetings and look at it in terms of the communication and all of the different access things that we talk about, like, go through them, categorize them . . .

M.C.: I’d find it interesting as well if we had a neurotypical person, just observing us to see what things would annoy them in meetings [. . .] I wonder what kind of things we do differently that we don’t even notice we’re doing, because we just accept it as an acceptable way of communicating [. . .]?

This article describes and reflects on the first six meetings of this community council, held over Zoom, involving the four community leaders and the first three researchers to join the project, all of whom are co-authors of this article. One aim for these first six meetings was to ensure the start of the project could be meaningfully shaped by Autistic stakeholder input, while co-developing accessible recruitment materials for the eight lay council members to come. Another aim was to cultivate a safe space and an initial model for fully accessible interaction within the community council. We consider the concept of a ‘safe space’ to be a space where those present are confident that they will not be harmed in any way or discriminated against. As our study activities take place in online meetings, this was primarily about the social aspects of the space. We reflect in the following sections on what made our community council feel safe and accessible. In this article, we draw on extracts from transcripts of recorded meetings, individual member ‘field’ notes and a reflexive co-writing practice (see the following sections). We explore how being a wholly Autistic team helped us quickly establish community connectedness and reflect on neurodivergent knowledge production.

## Our approach

With the growing prevalence of ‘out’ Autistic and neurodivergent academics and the establishment of collaborative and mutually supportive Autistic and neurodivergent scholar networks, there has been increased interest in how neurodivergent colleagues can work together to share interdisciplinary expertise, produce knowledge and create community, particularly so in the work of Bertilsdotter Rosqvist (2019) and Bertilsdotter Rosqvist et al. (2019, 2023a, 2023b). In their work, loose, collective accounts are iteratively woven together through a cooperative writing process referred to as *neurodivergent collective storytelling* (Bertilsdotter Rosqvist et al., 2023b) that seeks to find more neurodivergent-friendly ways of collaborating.

This article takes a similarly collaborative writing approach. In the first instance, we gathered data from the initial six meetings that took place prior to the addition of the further eight lay members in meeting seven. For the first two meetings, we had detailed notes and minutes. From meeting three onwards, the meetings had been recorded and transcribed. The video recordings were watched, and the transcriptions were annotated by the lead author. In our sixth community council meeting, we discussed how we would like to approach working together on this article and arrived at two key methodological questions:

1. How does the community council foster a sense of safety in which to comfortably express oneself, build community connectedness and facilitate neurodivergent knowledge production?
2. How is an inclusive, neurodivergent community of practice established and what does this look like?

We identified that most members would find a more open-ended reflective process unhelpfully unstructured. As such, members were sent a series of prompt questions (see Box 2) and asked to think about them or make notes in advance of one-to-one meetings scheduled with G.L.W. to discuss individual member’s experiences and perspectives. We all selected one-to-one meetings over Zoom as the most accessible means of working through our reflections, but these were explicitly not interviews. This predominantly conversation-based approach to data creation and analysis reflects our inclusive co-working practices and our effort to find ways of collaborating that work for us, even where they ‘trouble normative meanings of academic knowledge production’ (Bertilsdotter Rosqvist et al., 2019, p. 1082). Six one-to-one interviews were conducted and recorded (6 h 38 min in total), and G.L.W. collated key points into themes and a loose draft of this article that were revised collaboratively in a 2-h Zoom meeting and over subsequent emails.

**Box 2.** Prompt questions.

What came up in the one-to-one accessibility meeting you had with (Aimee: the primary investigator) before the first council meeting?
What did you ask for, how did you feel about asking, how did it go asking and how has it been actioned?
Is there anything else you’d like to add about your experience of being in the community council/any examples you want to give in relation to our two research questions?
How does working as part of the community council compare with other experiences you have of being involved in participatory or co-productive projects?

Here, we explore how we collectively forged an inclusive and specifically Autistic research praxis throughout these initial meetings. We reflect on neurodivergent ways of communicating and collaborating on this project that seem to go somehow beyond traditional CPPR. We ask ourselves the following question: Can we not only ‘cut our own keys’ (Bertilsdotter Rosqvist et al., 2023a) but also build our own house?

## Reflections on our emergent practice of neurodivergent knowledge production

**Figure 1.**
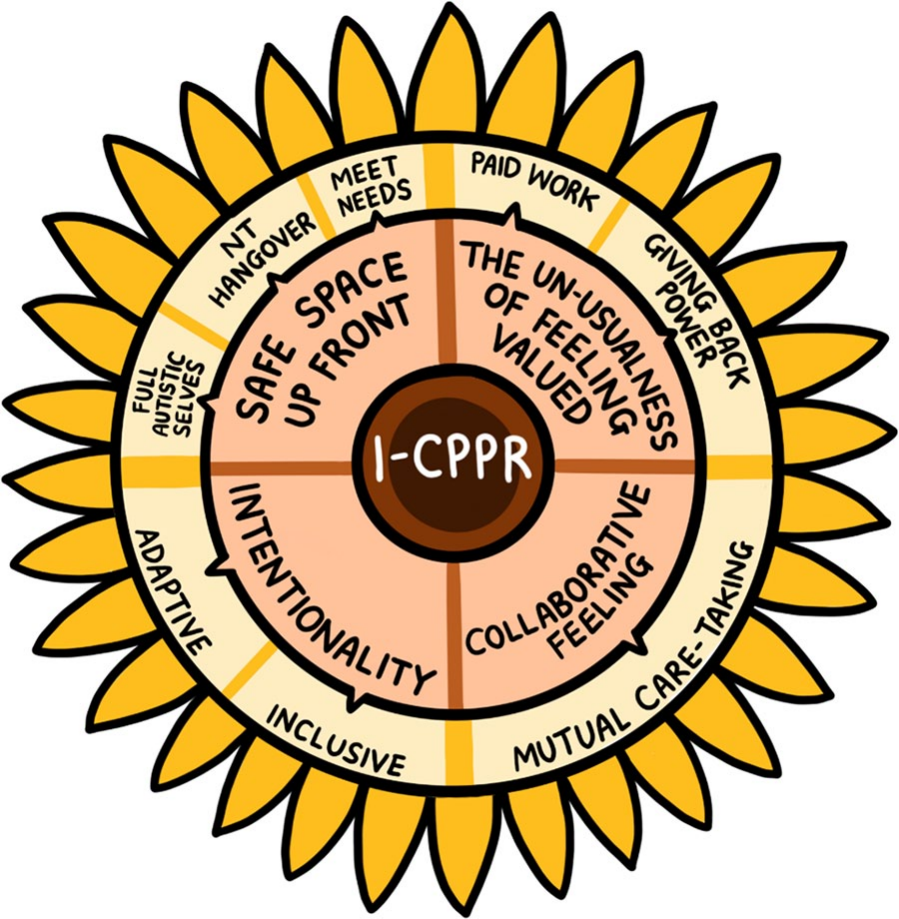
Graphical representation of the impact of Insider-only Community Partnered Participatory Research (i-CPPR) on neurodivergent knowledge production.

We organised our observations into four central themes: (1) *collaborative feeling*; (2) *intentionality*; (3) *creating and reiterating a safe space up front* and (4) *the unusualness of feeling valued*. These themes are interconnected and influence each other. We have represented these in Figure 1 *as parts of a sunflower* radiating out from our central praxis (*Insider-only Community Partnered Participatory Research – i-CPPR), which influenced our approach and led to the reported themes*. During theme development, we generated sub-themes which, although remain on our sunflower image as we consider them to be useful points of consideration, were subsumed, in most cases, into the related main themes. However, theme (3), *Creating a safe space up front*, is best thought of as a super-theme, comprising its three constituent parts (which we do discuss below: (1) *where we’re all accommodated and invited to ask for what we need*; (2) *where negative hangovers from working in NT spaces can be shaken off* and (3) *where we can bring our full Autistic selves*.

### Collaborative feeling

There’s a shared, mutual feeling that we are collaboratively building. (R.E.)

One of the most striking (though slightly ineffable) qualities of our community council that we have each reflected on during this process is a clear sense of *togetherness* (Williams, 2020, p. 321) that continued to grow over time. This included feelings of belonging, community and collaboration. This has shown up in the mutual care-taking of each other’s differing needs during meetings and an effortlessness in mutual understanding:

I think we are nurturing and understanding group of people that have come together to work on this project. We’re a rather empathic bunch despite what some other[s] might think. (K.W.)

From the very outset, we have perceived an evident ‘community collaborative ethos’, where:

we all want to hear each other’s opinions, no one wants to be the top dog. (W.H.)

It is possible, we have reflected, that some of the speed at which this was formed might be attributed to the fact that many of us already knew (or at least knew of) others in the group, meaning that we could already be sure of our shared values and predict some of our shared access needs:

I think it’s helped, because we’re actually quite a small community in Wales, and the community of advocates is even smaller, so all of us [. . .] even though we’re from different parts of Wales, we all know each other from before. This is probably the most intensely we’ve worked together, but we will have come across each other in different situations, so we already had some sort of idea of what each other’s needs were. (W.H.)

However, many of us have had experiences in groups previously where we have been familiar with the other members and shared their core values yet not found the same easy camaraderie and kinship:

I think that it’s helpful that everybody, that we kind of all know each other [. . .] I think that does help, but I don’t think it’s the only thing that’s done it [. . .] because I’ve also worked in other groups where I’ve known all of the members but it’s been more of a mixed neurotype and it has still had those difficulties or I’ve felt slightly on edge still. (K.W.)

It was through our discussions about this rare, equitable and collaborative feeling that we began to realise how unique being an *insider-only* CPPR council was.

### Intentionality

Our council has been founded with accessibility intentionally and explicitly baked in. This signalled to us from the outset that this was a project we could trust. Before the first meeting, all members were invited to meet one-to-one with the principal investigator to discuss accessibility needs:

Now for me, that’s a demonstration, that’s one of those little flags – you know like people would look for a little fish above a door to know if it’s a Christian house and it’s safe for them, or people might look for a little rainbow in the window of an establishment to know that it’s safe for LGBTQ people? So for me it’s one of those flags I think people look for, consciously, to know whether this is going to be a place where you can, basically, freely be yourself and they’re going to take your needs into account. (S.C.)

The council specifically aimed to be neurodiversity-affirming, that is, to allow everyone to contribute in the way that lets them do their best work (Naylor, 2023).^3^ In addition to signposting the neurodiversity-affirming stance of the council, these fact-finding meetings proactively preempted potential challenges, rather than waiting until we struggled before offering support:

The fact that we’re looking at issues before they happen: that’s the big one [. . .] and it’s giving me confidence. (M.C.)

The inclusivity of the council has also, crucially, been *adaptive.* At the beginning of every meeting, our chair displays on their screen and reads out our standard reminders (see Box 3). These were developed to serve as accessibility prompts and a mutually agreed community code for our interactions.

**Box 3.** Our standard reminders (as of meeting 6).

• It’s okay for everyone to have forgotten things and to need reminders for them.
• You don’t need to apologise if you’ve forgotten something.
• It’s okay to remind one another if something hasn’t been done.
• Becky will be monitoring the chat and if anything’s difficult for you feel free to message Becky privately.
• We’re all Autistic and it’s okay for us to behave in Autistic ways. This means stimming is fine, having breaks is fine, and anything else you need to do to be able to take part, is fine.
• If you feel overwhelmed, it’s always okay to leave. You can catch up later if you like, but don’t feel that you have to if it’s too much.
• Everyone will have the opportunity to contribute to each item and if you’ve thought about something you want to say about a topic that we talked about previously, it’s okay to go back to it and add your comments.

As the meetings went on, these standard reminders have evolved. Aware that they sometimes need memory prompts, for the first couple of meetings, A.G. had included ‘it’s always okay to remind me of something that I said I’ll do and I’ve not done’ as the first point. In meeting four, some of us suggested a change:

**Table:** 

Meeting 4: extract
*A.G.: So . . . [reads Standard Reminders]*
*S.C.: I do have something to add, it’s, I guess [. . .] because I noticed that it says, ‘it’s okay to remind you’ [i.e. AG] Okay . . . let me just figure out how to say it . . . It’s okay to have forgotten things that you need reminders for, like, I need it and I think practicing saying ‘thank you’ rather than sorry is a good thing for all of us. Do you know what I mean? Does that make sense?*
*K.W.: Yes, I know what you mean, it’s like, Point A [It’s okay to forget things] is on there but there’s always been an apology when it’s being used. So it’s almost like apologizing for something that is literally one of the things we’ve all agreed is okay. So no apologies needed.*
*S.C.: And I do think, you know, ‘thank you’ is a good thing. It’s like a ‘Oh, thank you for the reminder’. So, it feels more of a positive outcome. Does that make sense? It’s good practice.*
*W.H.: It’s ‘thank you for the reminder’ rather than ‘sorry I forgot’.*
*A.G.: Okay [. . .] so the first one is now ‘it’s okay for everyone to have forgotten things and need reminders for them. You don’t need to apologize if you’ve forgotten something.’*

**Table:** 

Meeting 2: extract
[Regarding S.C. being anxious about coming back into the Zoom room, late from a break:]
***A.G.**: . . . Would it be helpful for me to just reiterate at the beginning of meetings that it’s always fine to pop away for a bit as a lot of what we’re doing isn’t fast decisions so people can pick it up?*
***K.W.**: It’s okay if you’re late back or if you’re struggling to get in, it doesn’t mean that you’re going to be kicked out the group or not allowed to join that meeting. It is important, particularly when we have recruited the lay members to have that reiterated . . .*
***S.C.**: It’s very internalised, I know it’s okay, I know I’ve heard it said that I can come and go but I still beat myself up and that’s hard. So, I need the outside reassurance.*

While this adaptation did originally benefit A.G. – and help them address their own needs, which they found hard to keep in mind as the primary investigator, managing all our needs – it has ultimately benefitted us all. Many of us came into the council chronically over-apologising (see Theme 3b), and this gave us all permission to ask for what we need.

### Creating and reiterating a safe space up front

Something that has been patently evident to all of us is the sense of safety within these early meetings of our community council. This has been built through intentionally and reiterated from the outset, through being adaptable and inclusive, and by creating a sense of *we-*ness, as noted in the ‘Intentionality’ theme, with the inter-relatedness noted below:

Aimee and the other council members, they kept saying at the beginning of every meeting, middle of every meeting, end of every meeting: ‘This is a safe space. We are all neurodivergent’, you know, therefore, there’s this kind of like a shared baseline that we all have [. . .] a shared kind of unspoken understanding, that acts kind of like a foundation upon which we build. (R.E.)

The result of this feeling safe has meant that we have been able to ask for what we need and to shake off uncomfortable hangovers from being in neurotypical-dominant spaces where we have had to shrink, hide or contort our Autistic selves. In the following sections, we finish the statement ‘creating and reiterating a safe space up front’ through three sub-themes.

#### Where we are all fully accommodated and feel comfortable to ask for what we need

For many of us, being asked what we needed to make meetings accessible was something we had not experienced before:

I think the fact that there was [an accessibility meeting] in itself is something that I’ve never experienced before [. . .] People have said in a very stock way, ‘do you have any accessibility needs?’ [. . .] as a sort of tag-on in an email or something, but I’ve never had that sort of genuine ask. (K.W.)

I was able to tell Aimee, you know, it’s okay to text me to say ‘are you coming today?’ because there’s a good chance that I’ll genuinely have just forgotten. That’s something I’ve never been able to tell people in other meetings. (M.C.)

Importantly, democratising and valuing communication needs and modes have meant that nobody’s communication style is perceived negatively or as ‘impaired’, as it is often framed in neuronormative spaces (Williams, 2021). This has enabled everyone to feel confident to participate in whatever way they need, which can fluctuate:

We’ve very much allowed for a variation of communication methods in there. [It’s] recognised that actually some people are going to need you to meet with them first to give them a briefing of what the meeting’s going to involve, and that’s available if they need it. Recognising that a lot of us have got executive dysfunction, we’ve also got real problems with our memory [. . . and] reminders are put in place. One of the other massive things for me is that because I’ve got visual difficulties things like slides with a white background I just cannot see the text on it. And Aimee and the others in the project have been the only people ever who have not actually complained about doing it or have tried to do it with different colours because they don’t like the yellow. (W.H.)

However, juggling the dual role of insider–researcher presents unique challenges, and as Raymaker (2017, p. 268) has reflected, it involves the intersectional coexistence self, ‘as a whole person with overlapping facets’. For our Autistic chair, this was complicated further by the additional dimension of being the primary investigator on the project:

I’ve been doing lots of reading and thinking about how to make it more accessible, and it was literally only when I saw the prompt questions [Gemma] sent to me about, you know, what were my access requirements and how were they addressed that I . . . I didn’t even consider that I had the right to do that. (A.G.)

Reflecting further on this, our chair recognised that within the community council space, meeting the needs of members and ensuring that the meeting runs in a way that benefits the project and members was their priority. Reducing cognitive burden occurred through delegating some tasks that they found difficult, such as remembering to record the meeting and download the chat, to other members of the research team. However, ensuring her needs are met is something that largely comes outside of the meeting; for example, always thoroughly planning meeting activities in advance and not scheduling meetings before or after the council.

#### Where negative hangovers from working in neurotypical spaces can be shaken off

Many of us reflected that it took a little time, and some repetition, to deconstruct and shake off some of the unhelpful practices we had adopted from a lifetime of being socialised to meet neuronormative expectations, including when working in neurotypical-dominant settings. For some of us, we had to learn to stop apologising every time we spoke. For others, it was learning to trust that our tone or delivery would not lead to bad-faith interpretations. Nevertheless, this was something we all felt safe (and encouraged) to do and worked to support each other in achieving:

Removing the (neuro-)typical barriers to engagement and self-expression mean that members can better contribute to the project without needing to apologise for Autistic behaviours, ultimately generating richer impacts. More than that, it reduces the risk of harm caused from camouflaging (Cassidy et al., 2020) that many of us have previously had to endure:

What has been lovely is I’ve been in [other] meetings where I’ve come away feeling incredibly uncomfortable even though I’ve been able to mask it quite well in the meeting [. . .]. I don’t have that feeling in this group. (K.W.)

The whole of my working life has been shaping my working habits into a neurotypical mould. You develop coping mechanisms to help you in these circumstances so when you’ve been doing that for so long you don’t realise the cognitive effort it’s taking you to perform this way [. . .]. Then obviously, like it has in previous jobs, it leads to burnout and bad mental health. (R.E.)

#### Where we can bring our full Autistic selves

One of the big pros is that we are bringing our true authentic Autistic selves, experiences and opinions into this research. And therefore, all of you is coming in, whether you like it or not. (R.E.)

In a safe, insider-only space, we – both council members and researchers – quickly felt able to loosen our masking and test out how it might feel to show up in our full Autistic selfhoods, stims-and-all:

In other [groups], I tend to sit there, scared to move because my stimming can be problematic, scared to speak because people tend to take me the wrong way and I don’t get any of that with this group. (M.C.)

One of the big things for me – because I’ve grown up being told that I’m very blunt, that I need to be more careful with the way I choose my words and things like that – I over-explain myself [and . . .] I feel like I have to soften everything I say [. . .] because, again, being told [. . .] that my “tone is sometimes off” [. . .] I tend to feel quite uncomfortable [. . .] normally, even when it’s been with a few other Autistic people [. . .]. When I first started with this I had that worry and within, I reckon, two sessions, that worry was just gone completely. (K.W.)

I remember in one meeting [. . .] I thought I’d turned my video off. And I thought, right, I’ll try doing some stimming [. . .] but I hadn’t turned my camera off . . . and I was just there like, stimming along, and no one said anything or did anything and I had a moment where I was like, oh, okay, so that’s good to know. (R.E.)

Co-creating a space where individuals with marginalised and stigmatised identities feel safe to be ourselves is a form of emancipatory practice that truly models and enacts the project’s intentions to progress quality of life within the community:

This group is helping me not only feel like I’m okay to be myself – like truly okay, not just the pretend it’s okay – but it’s also helping me unpick all of [this . . .] internalised ableism. (K.W.)

### The unusualness of feeling valued and equal partners

Throughout this process, we have all reflected how unusual – yet welcome – it is to feel properly valued. This comes from being treated equally, rather than tokenistically:

That’s a key word I’d like to see in this [paper], that we’re all treated equally. I don’t ever feel that Aimee’s lording over us because she’s the lead researcher, or any of the academics involved. I actually feel like I’m completely in there, but we’ve achieved that through equity: by providing what people need to succeed. (W.H.)

Having put the stringent accessibility measures described earlier into place, members have been enabled to contribute meaningfully. It is possible for anyone to get accessibility right – by taking time to identify individual access needs and by dedicating sufficient resources to enact them. This often falls to one or more members of the team organising community councils, and in our case, the responsibility for ensuring access needs are met is primarily held by the lead facilitator, who ensures that needs are met by keeping and updating lists and referring to these prior to meetings and ensuring all elements are enacted. However, something about us all being *insiders* has meant that much of this comes intuitively:

Groups that been led by organisations, that [. . .] for example, have an Occupational Therapist that’s facilitating an Autistic-led group, they don’t have any of this [understanding . . .] so the dynamic is very different; we’re looked down on [. . .] When the group is just Autistic people we’re all treated as equals and everyone’s opinion is valid. (M.C.)

One further unusual (but welcome) aspect of being involved in this project is being paid adequately for our time:

[Here] we’re valued more than monetarily (but the money part matters too). (M.C.)

In our experiences, we have rarely been paid for our advocacy work. Many of us reflected on times we have given our consultation to projects or delivered presentations or training sessions without renumeration, often coming away feeling ‘othered’ or ‘disenchanted’ (Michael, 2021):

. . . my gripe is that when they [a well-known autism charity] invite me as a speaker, it’s never as a paid speaker because they don’t consider me a professional; they consider me a ‘person with’ [autism], and I don’t understand why ‘person with’ [autism] automatically means you don’t get paid. And the fact that their response is ‘we’re a charity’. My counter is: well put it in your grant applications. (S.C.)

Being paid – properly – for our time has demonstrated in action that we are valued and equal members. Autistic people are far more likely to experience financial hardship than non-Autistic people (Grant et al., 2023) and are often underemployed (current estimates put only 29% of Autistic people in work in the United Kingdom) or in severely insecure employment (38% of Autistic workers: Navani et al., 2023). In this context, not being paid for our *insider expertise* (Milton, 2014) feels especially egregious.

However, ensuring members were paid in a timely manner was not always easy. University systems are often not set up to pay community-based co-researchers (Williams, 2020), and bureaucratic hurdles meant delays in paying members for the first couple of meetings, meaning our Autistic primary investigator felt a personal responsibility to cover the gap:

. . . that is legitimately the sort of thing I will lose sleep over [. . .]. I said to everybody: if you’re struggling for money just let me know I can lend you the money until it comes in [. . .] You realise there’s a difference between me as a fully-salaried researcher and the Council members. (A.G.)

We do not believe that researchers should have to bear this burden individually. However, the fact that our primary investigator felt safe enough with the rest of us to offer it, and was willing, added to our sense of *togetherness* (Williams, 2020) and being valued, despite the inevitable power imbalances it reveals.

## Autistic tools for an autistic house (or, our ‘further discussion’)

From early on in our first meetings, we noticed a tangible difference in the quality of the space we were co-creating. We often commented on the fact that it felt like ‘more than co-production’ but could not, at first glance, identify what this difference was. While working on this article and developing our reflections, it became clearer that there was something important about the fact that we are all *insider researchers* and/or community members. As Raymaker (2017, p. 270) has highlighted, the absence of ‘insider perspectives in scientific inquiry’ presents ‘significant ethical problems’. The inclusion of insider (here, Autistic) researchers within the research team is one important way of addressing these. However, power dynamics and the position of privilege that come with being in academia complicate insider–researcher positionality, meaning that insider–researchers are not sufficient representation of community perspectives.

In traditional CPPR or CBPR (when it is done well), power is shared equally between community representatives and outsider-majority researchers. Different responsibilities are held by the different parties, for example, with academics, ensuring research remains scientifically sound, and for community members, ensuring research remains respectful and accessible (Nicolaidis & Raymaker, 2015). In our insider-only CPPR community council, with our mix of insider–researcher and insider community members, the boundaries between where these responsibilities lie are more blurred. Moreover, and crucially, *all* the power is held by insiders. Audre Lorde (1984/2007, p. 112) famously asserted that ‘the master’s tools will never dismantle the master’s house’. In working as an insider-only CPPR community council, we have not so much tried to *dismantle* the master’s house, but rather to build our own.

During these first six meetings, we perceived an unfamiliar sense of safety and depth of equity. This seemed to come from a feeling of *togetherness* (Williams, 2020, p. 321), or the knowing that we are all *One-Of-Us*, a term often used by Bertilsdotter Rosqvist et al. (2023a, 2023b, in press) to highlight the collectiveness of united neurodivergent voices, particularly when positioned as a counter to a dominant neurotypical voice within autism research. This early mutual trust may have been facilitated by the fact that many of us already knew each other or were aware of each other’s advocacy work. However, this was not the whole reason, and it is likely that the explicit and intentional approach to accessibility and mutual care-taking augmented this collaborative feeling.

By reiterating our community code and intentions for accessibility at the start of each meeting – in the form of what became our standard reminders (see Box 3) – our stance was made explicit and routinely reinforced. Moreover, it became part of our shared cognitive environment (and, ‘mutually manifest’, Williams, 2021), something we each declaratively knew, and knew was known by the others. Our code and stance became part of our shared common ground, and thus easier to act on.

As an *insider-only* council, we were also unhampered by the double empathy problem, not only because we are of similar neurotypes (Milton et al., 2018) but also because we all have lifelong experience of being misread or misrepresented, a common experience for Autistic individuals (Camus et al., 2022). This has perhaps meant we approach our group interactions with a greater generosity of interpretation:

We know how that feels and we know that we don’t want other people to feel that way. (K.W.)

Finally, it is important for us to reflect on the fact that we have received dedicated funding allocated to maintaining our 8-year community council. Without this funding, we would not have been able to ‘build our own house’ in the same way. For example, currently Aimee spends at least 1 day a week supporting the community council and often more than this. Our funding also pays for honorariums for community council members’ time, including preparation time, which for some includes additional one-to-one meetings with Aimee to review documents ahead of the meeting. Finally, our funder also pays for support workers’ time where council members would not be able to independently participate. We urge those developing funding applications to adequately charge for these items.

## Concluding thoughts

In this article, we have reflected on our experiences working together as an *insider-only* CPPR council. From the outset, we perceived a unique, collaborative feeling of *togetherness*, a sense of safety and the room and support for us to show up as our full Autistic selves. We have simply noticed an interesting and rewarding difference when working collaboratively in an *insider-only* space that has repercussions both in terms of community emancipation and research quality. Also important to note is that while community members were intentionally (and not randomly) selected, there was an element of luck in our council being an ultimately *insider-only* group. UK employment law does not allow for posts to advertise *only* for Autistic researchers, so the criterium used instead was that candidates be neurodiversity-affirming, something that non-Autistic researchers may also embody. As such, we do not mean to suggest that quality and inclusive autism research cannot happen when non-Autistic researchers are part of the team. Finally, we urge autism researchers to be mindful of their perspectives and privilege and suggest that authentically following CPPR principles is resource-intensive but can be extremely valuable.

## Notes

1. Where Community-Based Participatory Research (CBPR) is based in university settings, it is better described as Community *Partnered* Participatory Research (CPPR: Nicolaidis & Raymaker, 2015).

2. Not all people with wombs are women, and as such, we use trans-inclusive language throughout this article, reflecting our trans-inclusive stance to the project. The research focus of this project is on Autistic reproductive and gynaecological experiences, inclusive of Autistic people who *had* wombs and have since had hysterectomies.

3. This is also sometimes referred to as being neurodivergent-affirming or neuroaffirming.
